# Employés of the United States Bureau of Animal Industry at Buffalo, N. Y.

**Published:** 1900-11

**Authors:** 


### BUREAU OF ANIMAL INDUSTRY.—NOTES.

### EMPLOYÉS OF THE UNITED STATES BUREAU OF ANIMAL INDUSTRY AT BUFFALO, N. Y.

Dr. Charles H. Zink, inspector in charge; Dr. Bernhard P. Wende, inspector of cattle yards ; Dr. John P. O'Leary, inspector of sheep yards ; Dr. W. N. D. Bird, inspector of hog yards ; Dr. Harry M. Ball, inspector of export horses ; Dr. Clarence O. Durfee, inspector of abattoir 190 ; Dr. Louis A. Robinson, inspector of abattoir 42 ; Dr. Herman R. Ryder, inspector of abattoir 42 ; Dr. Alfred F. Martins, microscopist 42.

Beside the inspectors and assistant inspectors on the force, there are employed in stock-yard and abattoir work two clerks, four stock examiners, seven taggers, and nine ladies as assistant microscopists. The work at Buffalo is varied in its extent. Beside the yard inspection and abattoir inspection in 190 and 42, the tagging of export horses and mules, the dipping of diseased and exposed sheep for sale, tagging of export cattle, the microscopical inspection of export pork, there is the inspection of Canadian livestock at the various ports of entry in Buffalo, N. Y., Niagara Falls, N. Y., Suspension Bridge, N. Y., Lewiston, N. Y., and Charlotte, N. Y.

One of our co-laborers in the inspection force, Dr. Alfred F. Martins, who has charge of the microscopical inspection, has invented a very ingenious substage, which is attached to the microscope and is manipulated with a small crank, the turning of which causes the slide on which the specimen to be examined is placed to work up or down. After reaching a certain point a cog is passed, and the slide is slipped to one side, and proceeds back before the centre of the field of vision of the microscope. It continues backward and forward until past the field of vision. It then becomes unlocked, and another slide is placed in position, and the slide is pushed backward, locking itself in the operation, and can only be unlocked until the slide passes over the field of the microscope. This attachment is especially valuable in the examination of samples of pork, to detect the existence of trichinæ, as every portion of the slide passes the centre of the field of vision, and no portion escapes a careful scrutiny. The department has furnished all the microscopes at this point with this attachment, under the control of Dr. Martins, and he reports that in their use a much more satisfactory result is obtained.

Dr. Louis P. Green, a former employé on the Buffalo force, now in charge of bureau work at Detroit, Mich., was married on September 19, 1900, to Miss Emma Becker, an assistant microscopist on the Buffalo force.

Dr. Louis A. Robinson spent his vacation at Goderich, Canada. He was stationed at Detroit during the vacation period of Dr. Green.

Dr. Harry M. Ball sojourned in Ohio during his vacation.

Dr. B. F. Wende visited Cincinnati, Ohio, and Lexington, Ky. He said the vacation was not half long enough.

Dr. Herman H. Ryder took the right step on October 16th by taking a better-half.

